# Fingerprint of climate change in precipitation aggressiveness across the central Mediterranean (Italian) area

**DOI:** 10.1038/s41598-020-78857-3

**Published:** 2020-12-16

**Authors:** Nazzareno Diodato, Fredrik Charpentier Ljungqvist, Gianni Bellocchi

**Affiliations:** 1 Met European Research Observatory, International Affiliates Program of the University Corporation for Atmospheric Research, Via Monte Pino snc, 82100 Benevento, Italy; 2 Department of History, Stockholm University, 106 91, Stockholm, Sweden; 3 Bolin Centre for Climate Research, Stockholm University, 106 91, Stockholm, Sweden; 4 Swedish Collegium for Advanced Study, Linneanum, Thunbergsvägen 2, 752 38 Uppsala, Sweden; 5 Université Clermont Auvergne, INRAE, VetAgro Sup, UREP, 63000 Clermont-Ferrand, France

**Keywords:** Climate sciences, Environmental sciences, Hydrology

## Abstract

Rainfall erosivity and its derivative, erosivity density (ED, i.e., the erosivity per unit of rain), is a main driver of considerable environmental damages and economic losses worldwide. This study is the first to investigate the interannual variability, and return periods, of both rainfall erosivity and ED over the Mediterranean for the period 1680–2019. By capturing the relationship between seasonal rainfall, its variability, and recorded hydrological extremes in documentary data consistent with a sample (1981–2015) of detailed Revised Universal Soil Loss Erosion-based data, we show a noticeable decreasing trend of rainfall erosivity since about 1838. However, the 30-year return period of ED values indicates a positive long-term trend, in tandem with the resurgence of very wet days (> 95th percentile) and the erosive activity of rains during the past two decades. A possible fingerprint of recent warming is the occurrence of prolonged wet spells in apparently more erratic and unexpected ways.

## Introduction

Both natural and anthropogenic climate change can alter storm (rainfall) erosivity or the R-factor, i.e., the power of rainfall to cause soil erosion as defined in the Universal Soil Loss equation^1^ and updated versions of it^2–4^, due to the modification of rainfall patterns in time and space^5^. The oscillatory behaviour of extreme rainfall variability, and its associate erosive power, have huge impacts on agriculture^6^, hydrological processes^7^ and socio-economic dynamics^8^ across multiple spatial and temporal scales^9, 10^.

Advances have been made in recent scholarship towards the understanding of the dynamics of past and future extreme precipitation worldwide^11, 12^. However, traditional climate extreme indices and large-scale multi-model inter-comparison studies, used for future projections of extreme events and associated impacts, often fall short in capturing the full complexity of impact systems^13^. Simulations with state-of-the-art climate models show noticeable uncertainty in terms of internal climate variability^14^ and climate response^15, 16^. In addition, only low temporal resolution (e.g., monthly and seasonal) precipitation time-series are available for longer time-periods, which reflect our still incomplete knowledge, and an inability to reconstruct long time-series back in time of the spatial and temporal distribution, as well as the magnitude and driving mechanisms, of the R-factor^17^. Rainfall erosivity is not only important for the understanding of surface-process dynamics such as erosional soil degradation^17–19^, and other landscape stressors like flash-floods and landslides^20^, but its dynamics also offer an opportunity to detect the fingerprint of recent climate change—especially in the Mediterranean region—which is a particular sensitive region regarding climate variability and a “hotspot” of climate change^21^. For parts of this region, the ongoing trend towards more extreme precipitation is expected to continue in the coming decades, contributing to the uncertainty in the projected occurrence and intensity of extremes^22^. High-resolution and well-dated records are needed to understand the long-term hydroclimatic variability in this region, but the careful rainfall measurements on sub-hourly time-scales, which are necessary to obtain actual rainfall erosivity values according to the (R)USLE (Revised Universal Soil Loss Equation) methodology^4^, are not available prior to the digital instrumental period starting in the 1980s^23^.

In the absence of such detailed rainfall information, rainfall variability and its linkage to atmospheric pressure systems can be inferred from meteorological observations available for the Mediterranean region over the twentieth century^24^and even earlier back to mid-seventeenth century on a restricted regional basis^25^, but also from time-series of millennium-long hydrological extremes derived from documentary sources^26^. Long series derived from documentary data can help linking local and regional hydrological extremes to impacts^27^, assess climatic forcing features^28^, and recognise the climatic variability in hazard-exposed areas^29, 30^. Their analysis supports that climate can vary in response to natural processes such as those governing the occurrence of erosive rainfall and, thus, help us to understand present-day hydrological dynamics and improve projections of future changes^31^.

In the Mediterranean region, local to regional climate experiences a mix of gradual and abrupt shifts of precipitating systems, including erratically distributed erosive storms^32^. In fact, while northern Europe is primarily influenced by the North Atlantic storm tracks^33^, southern Europe is situated at the junction of major air-mass circulations, periodically arising from Atlantic, Mediterranean and Siberia influences^34^. Different meteorological factors can contribute to the growth and recurrence of extreme rainfall, but two components appear essential for generating heavy precipitation over the Mediterranean region: a high water vapour content in the atmosphere and triggering events originating from thermodynamic or dynamic processes^35^. Deepening over the warm waters Mediterranean cyclones mostly form around a few centres, with a dominating region in the Gulf of Genoa, where a slowly moving low pressure field (or Vb-weather pattern) can bring large amounts of rainfall^36^.

**Figure 1:**
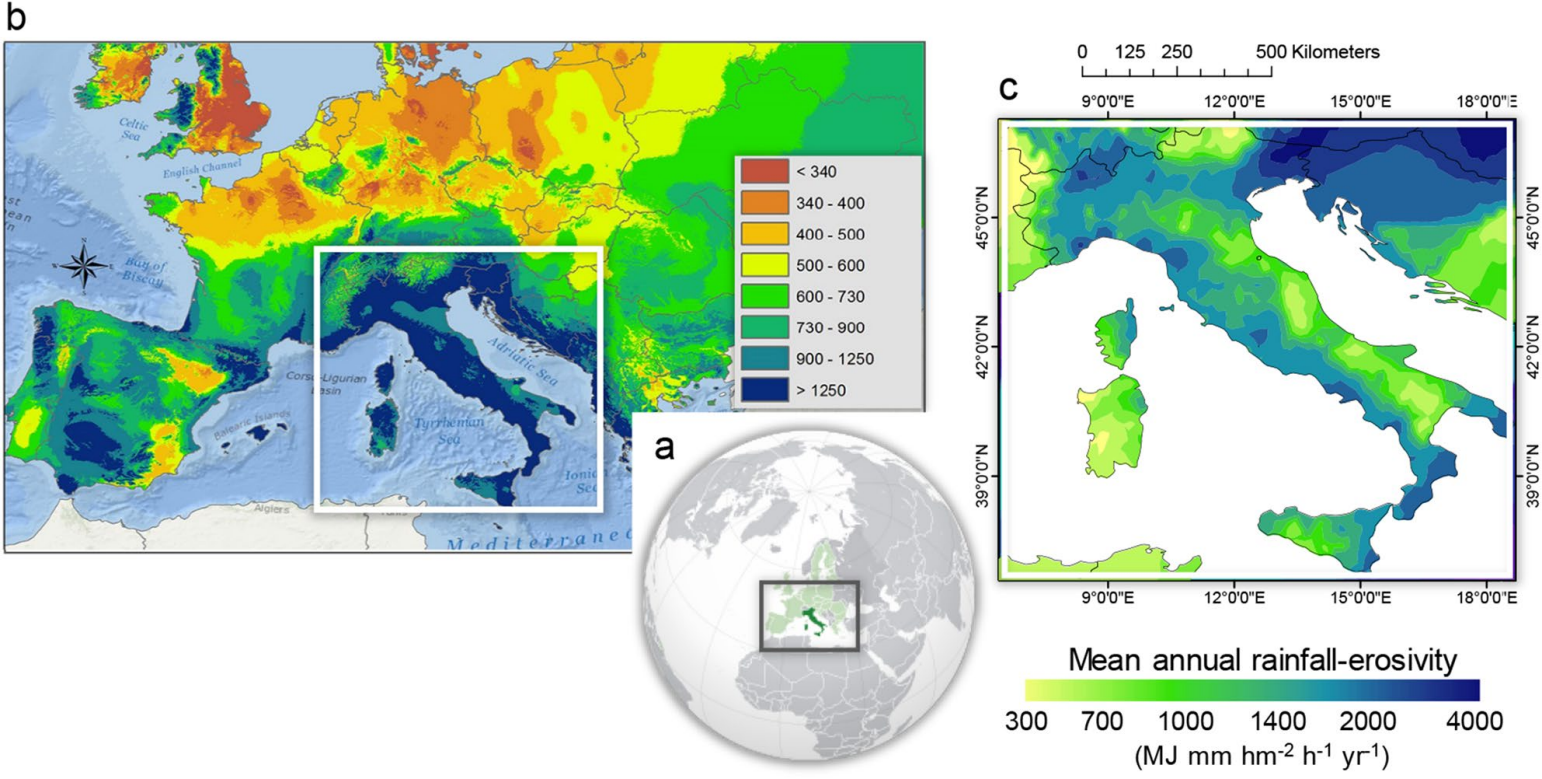
Study area. (**a**) Environmental setting. (**b**) Spatial pattern of mean annual rainfall erosivity (MARE) over central-southern Europe. (**c)** Map of MARE over Mediterranean Central Area for the period 1994–2013 (arranged with Geostatistical Analytics by ArcGIS-ESRI on the ESDAC-dataset, https://esdac.jrc.ec.europa.eu/content/global-rainfall-erosivity) ^55^.

**Figure 2:**
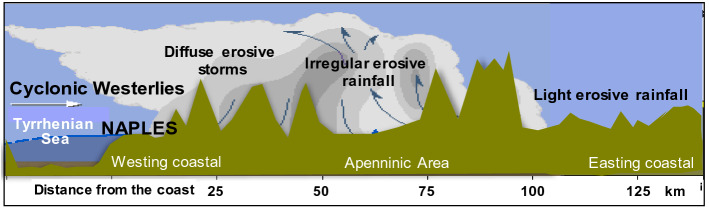
Scheme depicting the erosive rainfall pattern with cyclonic westerlies across a longitudinal transect west–east up to the middle Apennine of Italy. The city of Naples is shown, situated on the western coast of southern Italy (40° 50′ N, 14° 15′ E).

Modelling approaches making use of low-resolution precipitation data and documentary records of extreme weather events provide a means to derive long-term reconstructions. When satisfactory instrumental input data are unavailable, information from historical documentary sources can be used to support low-resolution storm-erosivity estimates^37^, both in time (i.e., with annual resolution or finer) and space (i.e., non-locally calibrated)^38^. In this study, we developed a modelling approach of the temporal fluctuations of storm-erosivity across the Mediterranean Central Area (MedCA), which is the most exposed region in southern Europe to aggressive rainfall (Fig. 1a,b). In this Mediterranean sub-region, high-intensity rainfall events can occur in any month due to the prevalence of thunderstorms throughout the whole of the year, which in turn can drive noticeable annual rainfall erosivity (Fig. 1c). On average for the period 2003–2012, rainfall erosivity over the MedCA ranged from over about 400 and 4000 MJ mm hm^−2^ h^−1^ year^−1^, with the western Tyrrhenian coast and the north-west being the most erosive-prone sectors with about 1500–2000 MJ mm hm^−2^ h^−1^ year^−1^ (Fig. 1c). In the eastern Alps and along the middle and low Adriatic (east) versant, the erosivity is somewhat lower, reaching values between 600 and 1000 MJ mm hm^−2^ h^−1^ year^−1^, respectively (Fig. 1c). Precipitation aggressiveness is generally triggered by synoptic disturbances coming from Atlantic cyclonic westerlies^39^, fed by heat and moisture fluxes from the Mediterranean Sea and maintained by mesoscale processes which, in turn, determine a suite of distinct, spatially-scattered erosive patterns (as in the schematic representation of Fig. 2).

Hillslopes are the dominant landform features in this area, where complex interactions between atmosphere and landscape systems operate at a range of time and spatial scales^40^. It follows that the mix of rain-producing erosivity events appears to vary not only spatially but temporally as well. However, detailed studies on the control factors of temporal dynamics are still limited, hence motivating the need of this study. In fact, some previous studies have determined rainfall erosivity using either limited rainfall records^41–43^ or non-uniform time intervals across different spatial scales^44^. Here, we used amounts of seasonal precipitation and weather anomalies to develop a simple, climatically interpretable model for reconstructing annual erosivity data in the MedCA over the period 1680–2019 CE. For both its length and spatial extension, this provided an unprecedented time-series to discern the climate change fingerprint and communicate the regional erosivity hazard in Central Mediterranean. In this study, we have excluded all data prior to 1680 because precipitation data for earlier times have been reconstructed from non-instrumental data exclusively. They are, thus, affected by a larger uncertainty than more recent instrumental data, especially for the southern sector of the MedCA^45^.

## Results and discussion

### Rainfall erosivity model calibration and validation

To estimate areal-mean annual rainfall erosivity over the MedCA, a simplified statistical model was developed, which summarises the relationship between spatial patterns of climate and storm erosivity, consistent with a sample calibration (1994–2015 CE) and validation (1981–1993 CE) of detailed (R)USLE-based data obtained for the study area. We calibrated the Rainfall Erosivity Mediterranean Model (*REMM*, Eq. (1) in Methods), based on multiple observational time-scales, such as: seasonal precipitation, its variability and the Gaussian-filtered annual severity storm index sum SSI(GF) from Diodato et al.^26^. For the calibration period 1994–2015 CE, we obtained the coefficients *A* = 0.0512 MJ mm^−1^ hm^−2^ h^−1^ year^−1^, *B* = 0.1128 MJ hm^−2^ h^−1^ year^−1^, *C* = 637 MJ hm^−2^ h^−1^ year^−1^, α = 10.0 and *k* = 6.0 in Eq. (1).

**Figure 3:**
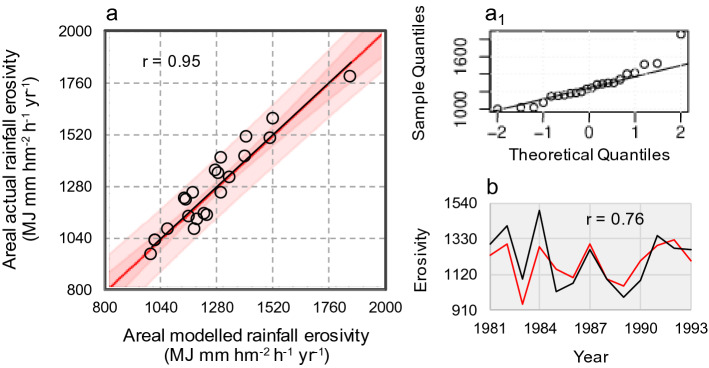
Model calibration and validation. (**a**) Scatterplot of regression model (black line, Eq. (1) and red line of identity) vs. actual rainfall erosivity estimated upon the MedCA over the period 1994–2015, with the inner bounds showing 90% confidence limits (power pink coloured area), and the outer bounds showing 95% prediction limits for new observations (light pink). (**a**_**1**_) related QQ-plot of residuals at calibration stage. (**b**) Time co-evolution (1981–1993) of actual (black curve) and estimated (red curve) of rainfall erosivity at validation stage (in **a**,**b**, r stands for linear correlation coefficient).

With these values, an ANOVA test returned a highly significant relationship (*p* ~ 0.00) between observed and predicted erosivity values. The R^2^ statistic (square of the correlation coefficient in Fig. 3a) indicated that the *REMM* explains ~ 90% of the erosivity variability. MAE (mean absolute error) was equal to 55 MJ mm hm^−2^ h^-1^ year^−1^—which is ~ 3% of the mean erosivity over the period 1994–2015—with the Kling-Gupta Efficiency (KGE) equal to 0.89. The calibrated regression (Fig. 3a, black line, Eq. (1)) shows only negligible departures of data-points from the 1:1 identity line (red line), indicating that the ability of the model (Eq. (1)) to predict actual erosivity in the MedCA is satisfactory. In particular, the regression line has an intercept a = –5.2 (± 95.6) and a slope b = 1.0 (± 0.1) near or equal to the optimum values (a = 0 and b = 1). Though the relatively high standard error of the intercept (± 95.6) indicates the model’s lesser predictive ability for near-zero erosivity values the intercept is not statistically different from zero (Student-t P ~ 1.0). The Nash–Sutcliffe efficiency value obtained in the calibration stage (EF = 0.9) also indicates limited uncertainty in model estimates. The distribution of the residuals approaches the normal distribution shape, indicating skew-free distribution of errors as the data points are mostly aligned along the QQ-plot function (Fig. 3a1). For the Durbin–Watson (DW) statistics (DW = 2.76), there is no indication of serial autocorrelation in the residuals (*p* = 0.97).

For the period 1981–1992, the ANOVA *p*-value less than 0.05 means that a statistically significant relationship between the estimated and actual data is maintained with the independent dataset used for validation. Though the R^2^ statistic (square of the correlation coefficient in Fig. 3b) indicates that the model only explains ~ 58% of the variability of actual erosivity, the time-variability as a whole was well reproduced (Fig. 3b). The regression parameters a = − 55.4 ± 322.8 and b = 1.1 ± 0.3 indicate that some model overestimation of near-zero erosivity data can occur with the model but the Nash–Sutcliffe efficiency value equal to 0.6 corroborates that also at the validation stage the uncertainties associated with model estimates are not large. The MAE was equal to 84 MJ mm hm^−2^ h^−1^ year^−1^—which is ~ 5% of mean erosivity over the period 1981–1993—while KGE was 0.5. This analysis also indicated that the categorical storm index SSI(GF) was a reliable substitute of storm rainfall. The importance of including this input in the model was confirmed by the values of r, which increased from 0.88 to 0.95 at calibration stage, and from 0.36 to 0.76 at validation stage. Also the mean absolute percentage error (MAPE), which measures the prediction accuracy of a model, indicated an increased accuracy, from 6.3 to 4.3% at calibration stage, and from 9.4 to 7.0%, at validation stage, when including SSI(GF) as input.

### Historical rainfall erosivity reconstruction

**Figure 4:**
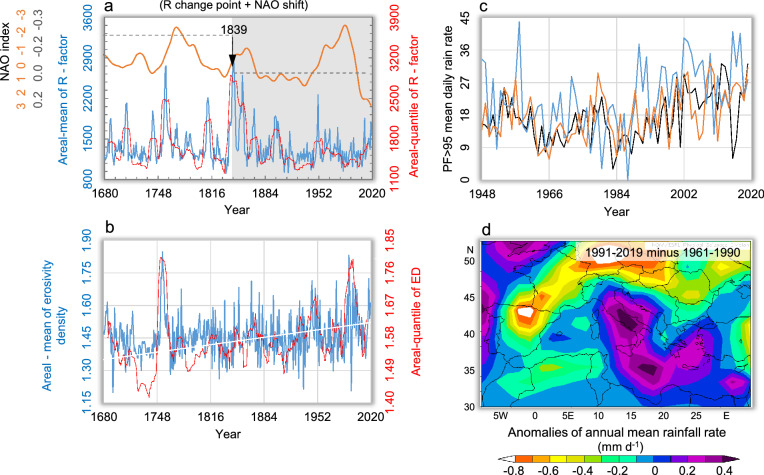
Overview of several precipitation patterns over MedCA. (**a**) Timeline evolution of annual rainfall erosivity reconstructed by Eq. (1) (blue curve) and its quantile with return period (RP) = 30 years (red curve) in a 21-year running window (in MJ mm hm^−2^ h^−1^ year^−1^) and with the related plynomial trend (white curve) after the change-point. Horizontal grey dashed lines are mean values of the North Atlantic Oscillation (NAO) for two periods (reversed axis), calculated from the proxy-based multidecadal winter NAO reconstruction of Trouet et al.^100^ (orange line). (**b**) Erosivity density timeline (blue curve) with its quantile with RP = 30 years during the period 1680–2019 (in MJ mm hm^−2^ h^−1^ year^−1^). (**c**) Precipitation fraction due to very wet days (PF > 95th percentile) for the MedCA (black curve), northern Italy (orange curve) and Sicily (blue curve) over the period 1948–2019 (in mm d^−1^). (**d**) Anomalies in the spatial pattern of annual mean rain rates (1991–2019 minus 1961–1990) over the Mediterranean region (from NCEP/NCAR reanalysis data, https://psl.noaa.gov/cgi-bin/data/getpage.pl).

Figure 4 shows the areal-mean erosivity evolution over the period 1680–2019 CE, as obtained by means of Eq. (1). The time-series was analysed to find out possible patterns of rainfall aggressiveness and to compare contemporary conditions with historical erosivity patterns (Fig. 4a). The long-term mean value of estimated erosivity data is 1367 ± 319 (SD) MJ mm hm^−2^ h^−1^. The application of the Standard Normal Homogeneity Test for the double shift^46^ (which is useful for dividing long time-series into shorter periods) suggests discontinuities (change-points) in the annual erosivity time-series at a 99% confidence interval in the years 1839 and 1858. The fingerprint of climate change was also detected with the quantile erosivity data with 30-year return period (red curve), with change points in 1838 and 1861, not dissimilar from those obtained with the erosivity data time-series. Other test statistics^47–52^ detected change points in 1873 and 1903 for the erosivity data, or 1876 and 1906 for the quantiles, which merely support the idea of a long transition period going from the final phase of the Little Ice Age (LIA; ~ 1300–1850 CE^53^) to the most recent warming. These different statistically-relevant years provide a loose picture of climate-related erosivity variations with changing climate patterns, where the cold conditions of the LIA are still dominating after the end of the Dalton minimum of reduced solar activity (~ 1790–1830 CE) until towards the end of the nineteenth century, but in the process of evolving into an incipient warming that becomes noticeable later in the twentieth century. Following the first detected change point, the quantiles’ values evolve according to a second-order polynomial, whose descending portion passes from 2500 to 1900 MJ mm hm^−2^ h^−1^ between the change-point around 1839 and 1950s (white curve on grey band). Our analysis supports a relation between our output (rainfall erosivity) and the dynamics of the North Atlantic Oscillation (NAO)^54^. The NAO represents a redistribution of air masses between sub-tropical (Azores archipelago, roughly 38°N) and sub-polar latitudes (Iceland, roughly 65°N), and modulates the strength and latitudinal location of the westerly flows, whose major influence on Central Mediterranean precipitation is documented^55, 56^. Both the positive and negative phases of the NAO are associated with regional changes in large-scale modulations of zonal and meridional heat and moisture transport patterns^57^. In particular, the positive phases of the NAO reflect lower than normal heights and pressure in the high latitudes of the North Atlantic, and higher than normal heights and pressure in the central North Atlantic and Western Europe (with negative phases reflecting opposite patterns). Strongly positive phases of NAO are usually associated with below-average temperatures and precipitation in southern Europe, while opposite anomalies of temperature and precipitation are generally observed during strongly negative phases. While the NAO shows considerable inter-seasonal and interannual variability, the winter NAO also shows considerable multi-decadal variability^58^. For instance, the negative phase of the NAO dominated the Atlantic circulation between the mid-1950s to the 1970s. An abrupt transition to recurrent positive phases of the NAO then occurred during the winter 1979–1980 (with the atmosphere still locked into this mode during the winter 1994–1995), followed by a return to a strongly negative phase of the NAO^59^. Figure 4a shows that phases in the studied period (1680–2019) turning to substantially neutral (with no clear dominance of positive or negative episodes) NAO state (0.12 ± 0.09 standard error, horizontal grey dashed line after the change point) correspond to a decline of the erosivity quantile.

We derived that the interannual variability of rainfall erosivity is pronounced either before or after the change-point (Fig. 4). However, the MedCA appears to be subject to more frequent peak values before the break-point (e.g., in 1707 with 2095, in 1757 with 2725, in 1758 with 2760, and in 1812 with 2126 MJ mm hm^−2^ h^−1^), a period dominated by the negative mode (− 0.40 ± 0.06 standard error) of the NAO.

This is in line with results from previous studies showing that the erosive power of rainfall and extreme precipitation is stronger during cyclonic (low-pressure) conditions (for the western Mediterranean^60^; and for Montenegro, on the east side of the MedCA^61^). Hydrological extremes may persist and evolve in unexpected and erratic ways; in fact, the estimated rainfall erosivity indicates a recovery in the low-frequency erosive activity of rains during the past two decades. It is difficult to detect signals of climate change in erosivity extremes associated with torrential rainfall (e.g., hourly-long events), due to the wide spatial variability and unpredictability of these events in long time-series. We addressed this issue by examining the erosivity density (ED), i.e., the erosivity per unit of rain, which is a better indicator of the climate erosive hazard than rainfall erosivity^31^.

The evolution of ED is shown in Fig. 4b, and it is surprisingly characterized by a significant increasing linear trend. The related ED-quantile (RP = 30, red curve) reveals a continuous and strong growth, with a significant long-term linear trend (Mann–Kendall test, *p* < 0.01). In this case, ED-quantile increases from 1.2, at beginning, to 1.4 MJ hm^−2^ h^−1^ year^−1^, at the end of time-series. Since both erosivity and erosive density appear subject to a complex evolution over the most recent (warmest) decades.

We inspected the period 1948–2019 in more detail by arranging the precipitation fraction (PF) due to very wet days (PF > 95th percentile) from the NCEP/NCAR Reanalysis data. We found that the PF > 95th percentile improves the accuracy of heavy precipitation estimates of very wet days to total precipitation from the probability distribution of daily precipitation than from the raw data^62^. In this way, we detected a temporal development of the P > 95 with an intensifying trend during the recent warming phase, 1986–2019, at both regional and sub-regional scales, confirming how the landscape, during recent decades, have been recurrently subjected to gradually increasing hydrological stress (Fig. 4c). Black curve in Fig. 4c illustrates the evolution of FP > 95 over the MedCA, while blue and orange curves represent the FP > 95 for northern and insular Italy (Sicily), respectively.

The anomaly map furthermore suggests an enhanced hazard associated with complex and more intense rainfall events over the MedCA during recent decades (Fig. 4d). It is characterised by a strong convective component, especially in the autumn season^63^. As a consequence, disaster-affected areas in the MedCA have become more exposed to climate hazard conditions because rainfall aggressiveness has apparently become increasingly changeable and unpredictable at small scales^64^. According to Cislaghi et al.^65^ and Pavan et al.^66^, the frequency of occurrence of daily precipitation has decreased over Italy, but short-duration episodes (i.e., from 1 to 3 h) have instead enhanced the torrential character of seasonal rains. Colarieti Tosti^67^ also reported that in the coming decades the polar vortex would likely go through a phase of expansion towards the southern latitudes, with consequent exacerbation of the hydrological cycle in the Mediterranean. An increasing frequency of extreme precipitation events is also expected over hazard-exposed landscapes over much of Europe towards the end of the twenty-first century^9, 68^.

Climate model simulations with the new generation of Coordinated Downscaling Experiment over Europe (EURO-CORDEX^69^) reveal an increasing trend towards a higher frequency of hydrologic extremes across most of Europe with future global warming. Towards the end of the twenty-first century, the results from the EURO-CORDEX simulations show for most European countries—including southern Europe—no significant change in annual precipitation, but at the same time an increase in maximum daily precipitation^70^. These results seem to reflect the paradoxical increase of Mediterranean extreme rainfall in spite of decrease in total values, as claimed by Alpert et al.^71^, Paxian et al.^72^ and Caloiero et al.^73^ but questioned by Mariani and Parisi^74^ for the whole basin and the western sub-basin. Other researchers^75^ also indicated no significant trends in the observed data, testifying a substantial stability of the temporal and spatial behaviour of heavy rain events over the course of the twentieth century. These contrasting results highlight that different analyses can change perception of the type of hazard associated with hydrological processes when using dissimilar metrics. With this study, we advocate the use of metrics that not only reflect the climate forcing component reproduced in the prevailing storm aggressiveness (erosivity), but in addition the damaging hydrologic hazard. The erosivity density offers this opportunity because regions with high ED values are exposed to a risk of flooding (and even water scarcity) because of their infrequent, but very intense and erosive rainstorms^76^.

While for the twentieth century, individual time-series of erosivity data are available at some Italian sites, a reconstruction was lacking that would enable a comparison between recent and historical erosivity covering particular climatic periods like the LIA. For that, we have developed and assessed a historical model (traced back to the seventeenth century), which can explain the erosivity data made available from previous studies. In this way, we have framed the historical trend of erosivity (with a view to exploring the transition from the LIA to the modern warming) rather than reconstructing with an arguable level of confidence the erosivity in each single year. Our work reveals that improved knowledge of the temporal changes in erosive precipitation is critical since these changes indicate different degrees of landscape damages and are of great importance for the implementation of environment conservation and management plans under a changing climate^77^. The occurrence of erosivity events is expected to be significantly altered under a warming climate. Since southern Europe landscapes tend to react rapidly to such changes, understanding extreme precipitation and the associated erosive conditions is vital to better comprehend and anticipate environmental changes in this area. Meeting the demand for reliable projections of extreme, short-duration rainfall is challenging because of our poor understanding of past hydroclimatic dynamics and mechanisms, which limits our ability to generate plausible predictions about future states^11^. As we have shown in this study, precipitation and storm indicators provide new opportunities to develop long records of past erosivity data. We improved the understanding of hydrological extremes, and the inter-annual variability of erosive rainfall and erosivity density, employing long continuous time-series covering 1680–2019 CE, developed for the MedCA from available instrumental and documentary climate records. The integrated approach used to reconstruct and assess yearly erosivity data allowed detecting signals of present-day climate change. For the entire assessed period 1680–2019 CE, the main conclusions can be summarized as follows:

1. Annual rainfall erosivity data across the MedCA from 1680 onwards show alternate stormy and quieter periods, with a change point towards the end of the Little Ice Age (1839) and different trends before (1680–1838 CE) and after (1839–2019 CE) that break point. Conversely, erosivity density has undergone an increasing trend since the beginning of the series.
2. The generally cold period 1680–1838 CE appears characterized by some peaks of stormy years, followed by a quieter phase, marked by a transition to decreasing storminess over the dominant mode of neutral NAO. During recent decades, however, the precipitation fraction due to very wet days (> 95th percentile) and rainfall erosivity indicate a recovery in the erosive activity from more intensive rainfall, concurrent with an increased variability of erosive density.
3. The way how in the recent decades Mediterranean cyclones have been producing trends of erosivity from rainfall extremes remains elusive^24, 25^. Whether cyclone occurrences decreased over the Mediterranean, a rising erosivity associated with short-term and very extreme rain events could also be due to a strong increase of extreme related convective-precipitation during recent warming. Berg et al.^78^ showed that convective precipitation is more responsive to temperature increases than frontal passages, and increasingly dominates rain events of extreme intsensity. Chains of damaging events would likely become more common with increasing climate warming^79^, though with distinct patterns of change in small domains without the emergence of spatially and temporally homogeneous trends^80^.

The present analysis, performed at an annual temporal scale, may mask important variations manifesting at even finer time-scales. The methodology applied mostly addresses inter-annual and inter-decadal time-scales, and does not capture daily to seasonal changes, which may impact on hydrology and lead to damages due to losses of land at different scales. For the MedCA, large erosive rain events were observed to occur especially in summer in continental areas, and in autumn along the coasts and near-to-coast reliefs^81^. It was also shown^82^ that more frequent extreme events in autumn do not cause seasonal rainfall totals to deviate from the historical range of climate variation. They rather tend to generate more disproportion between dry and wet periods, which could bring soil loss to higher rates^83^. The regional analysis may also obscure sub-regional trends^84^. However, only a few sub-regional studies extend back erosivity data over such a long period as in our study. Erosivity time-series reconstructed in two Mediterranean fluvial basins, the Calore river basin (southern Italy)^85^ and in the Po river basin (northern Italy)^86^, showed similar increasing trends since the end of the LIA. Diodato et al.^87^ also showed an upward trend in the Swiss plateau (north of the Mediterranean central area). If this rainfall regime would continue, it could result in an ever-increasing erosive hazard affecting Mediterranean lands in a more erratic fashion. This implies that a future increase in extreme precipitation events might have severe consequences regarding soil erosion, flash-flood risk, and various types of ecological disruptances in the Mediterranean region^88^. Furthermore, this underlines the need for high-resolution climate model projections to reliably predict erosive precipitation.

In conclusion, by providing the first quantitative assessment of the long-term dynamics of storm-related extremes in the MedCA, this work highlights the promise of this approach and provides impetus for further refinement of erosive rainfall reconstructions. The time-series of annual erosivity and erosive density presented here are arguably of great importance for climatic analyses dealing with climate variability at multidecadal to centennial time-scales. It is on precisely these time-scales that the anthropogenic-forced changes are most likely to be superimposed on natural (forced and unforced) climate variability. The erosivity data presented here can be used to study the variability and the extremes of climate occurring at a sub-regional scale, which can be compared to model-based simulations of natural and forced (external and internal) variability for the past centuries. This study also indicates the importance of erosivity density for representing and predicting future hydrological changes across the Mediterranean region. The limited availability of basin-wide consistent erosivity data constrained the ability of our study to comprehensively model erosivity for the entire region. Furthermore, with this limitation in mind, these results provide essential information to shed light on the processes governing long-term storm-related phenomena. This implies that environmental management can use data from long historical time-series as a basis for increasing societal resilience, informing decision making and directing new monitoring/modelling efforts. In particular, they can raise the awareness of policymakers by pointing to the urgent need of minimizing and controlling the land degradation in the Mediterranean basin. Finally, our study emphasises the importance of separating low-frequency trends from high-frequency extreme events that, as revealed here, may show entirely opposite trends. The inability of most early instrumental time-series, and climate reconstructions, to capture high-frequency extreme events have clearly limited the possibility to fingerprint anthropogenic climate change against the full range of long-term natural variability across time-scales. Whereas the mean state, and rate of change, for a particular climate variability may still be within the range of natural variability, the amplitude or return rate of certain climate extremes may not.

## Methods

### Study area

The MedCA has a roughly rectangular shape centred on Italy, with a preferential north–south orientation (36°–47° N, 5°–18° E). It extends over large portions of southern Europe, in the transition zone between Central Europe and the southern Mediterranean rim. The Alpine sector forms an imposing mountain range to the north, with a length of ~570 km and a width varying from 25 to 110 km. The Apennine mountain chain runs over ~ 1000 km along the Italian peninsula. The Italy islands of Sardinia and Sicily close this geographical variety. The climate of the MedCA is highly diversified, given its geographical position and the varied morphology of the sectors composing it. The meteorological conditions largely depend on the fronts arising as a result of cold polar air meeting warm tropical air. In particular, different situations are produced depending on the cell that is interposed between the subpolar cold air and the warm Mediterranean air on one side, and between the damp maritime climate of the west and the dry continental climate of the east on the other. The MedCA is subject to pronounced rainfall erosivity in the context of southern Europe.

### (R)USLE-based actual rainfall erosivity data

**Figure 5:**
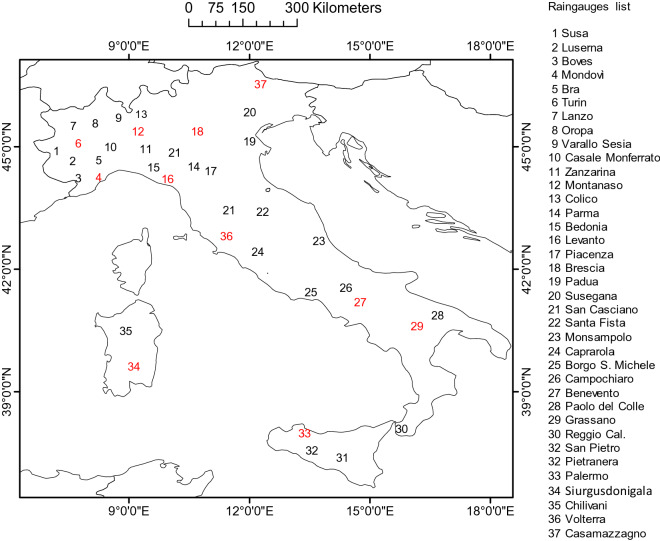
Spatial distribution of digital rain gauges used for calibration (black numbers) and validation (red numbers) across the Mediterranean Central Area.

**Figure 6:**
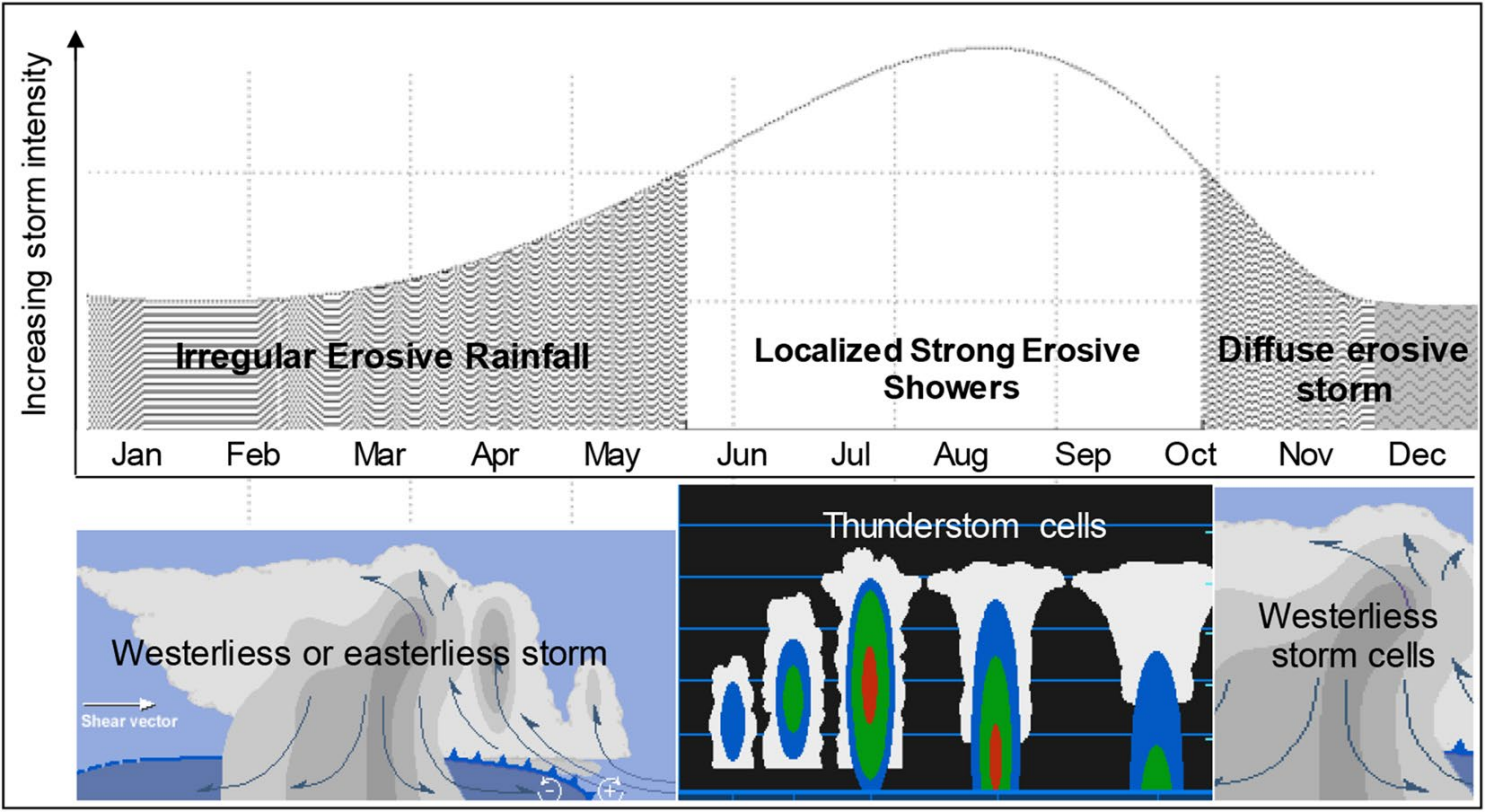
Seasonal regime of storm cells with the related scheme of variability of the monthly storm erosivity strength over the MedCA.

Annual (R)USLE-based R-factor data (1981–2015) were derived from Diodato^89^ and Acquaotta et al.^90^, with updating from the network of SCIA (http://www.scia.isprambiente.it/wwwrootscia/scia.html)—National System for the Collection and Elaboration of Climatological Data^91^, for a total of 37 stations (Fig. 6). For the period 1994–2015, (R)USLE-based data were available for the full set of meteorological stations (Fig. 6, both black and red numbers), because the digital National Agrometeorological Network (https://tinyurl.com/h4juzuv) came into operation in 1994. Prior to 1994, only some data were available for the Piedmont Region of Italy (north-western part of the MedCA), with only few sparse stations providing data since 1981. The most accurate dataset, 1994–2015, was used for model calibration, while the least accurate dataset, 1981–1993, was used for model validation (Fig. 5, red numbers).

### Numerical and categorical inputs

The reconstruction of annually-resolved rainfall erosivity for the MedCA was based on seasonal precipitation data covering most of Europe (30° W–40° E/30° N–71° N) from 1500–2000 on 0.5° by 0.5° grid resolution^92^. The GPCC (Global Precipitation Climatology Centre) provides an extension of the seasonal precipitation dataset until 2019 (retrieved from https://tinyurl.com/wlnvznq). GPCC products are known to outperform other similar products (e.g. CRU, ERA‐Interim, ERA‐40) in Mediterranean areas^93, 94^.

A categorical variable, the annual severity storm index sum (*SSI*) from Diodato et al.^26^ was used as a proxy to overcome the lack of reliable information about historical rain intensity. *SSI* was derived from several written sources by transforming documentary information into a record set to 0 (*normal event*), 1 (*stormy event*), 2 (*very stormy event*), 3 (*great stormy event*) and 4 (*extraordinary stormy event*). The *SSI* time-series was smoothed by applying the low-pass Gaussian filtering technique.

### Rainfall erosivity mediterranean model

For the historical reconstruction of annual rainfall erosivity (MJ mm hm^−2^ h^−1^ year^−1^), we developed a regression model, hereafter referred to as the Rainfall Erosivity Mediterranean Model (*REMM*), which uses seasonal precipitation data (mm) and the Gaussian-filtered Annual Storm Severity Index (*SSI*(GF) as inputs. The non-linear dependence of rainfall erosivity on rain intensity supports the adoption of nonlinear indicators within a parsimonious approach comparable to the (R)USLE approach^4^. The non-linear model takes the following form:

1 $$REMM=A\cdot {{\frac{SD}{Max(Ps)}\cdot P_{Sum}}\cdot P_{Aut}}+B\cdot \sqrt{{\mathrm{\alpha }+ ASSIS(\mathrm{GF})}^{k}}\cdot \left({P}_{Win}+{P}_{Spr}\right)+C$$

where *A* (MJ mm^−1^ hm^−2^ h^−1^ year^−1^) and *B* (MJ hm^−2^ h^−1^ year^−1^) are scale parameters converting the result of multiplications into the output unit, and *C* (MJ mm hm^−2^ h^−1^ year^−1^) is a shift parameter estimating rainfall erosivity when the seasonal precipitation (*P*, mm) inputs (*Sum*: summer, *Aut*: autumn, *Win*: winter, *Spr*: spring) are equal to zero. The intensity of the product between summer and autumn precipitation (${{P}_{Sum}\cdot P}_{Aut})$ is modulated by a modified version of the variation coefficient, where *SD* is the inter-seasonal standard deviation and *Max*(*Ps*) is the maximum seasonal precipitation per year (mm). The product (${{P}_{Sum}\cdot P}_{Aut})$ was considered here because the power of rainfall exerts the maximum of erosive forces in summer and autumn (Fig. 6).

Severe convective erosive storms occur between June and November in Europe^32^. For this region, the occurrence of severe thunderstorms is indeed intimately associated with the convective environmental conditions. The frequencies of flood and flash-flood events shows that the precipitation in spring likely leads to a different regime of hydrological extremes compared to June–October, when flash-floods are more frequent^32^. This means that the period June–November delineates a key time window in estimating rainfall–runoff erosivity in Europe.

The sum of winter and spring rainfalls ${(P}_{Win}+{P}_{Spr})$ is instead modulated by the storm index SSI(GF) within the φ term $\sqrt{{\mathrm{\alpha }+ ASSIS(\mathrm{GF})}^{k}}$. The central Mediterranean, although located to the south of the main Atlantic storm track that more directly affects northwestern Europe, is quite frequently subject to sudden events of extreme and adverse weather, which cannot be accounted for by only seasonal precipitation totals^26^. The additive component $\left({P}_{Win}+{P}_{Spr}\right)$ supports the assumption of a softer dependence of erosivity on precipitation intensity.

**Figure 7:**
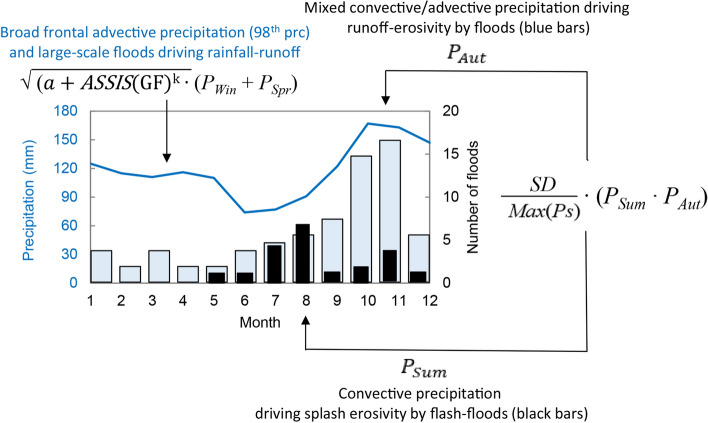
Monthly precipitation (98th percentiles) and frequency of floods and flash-floods (bars) driven by dominating frontal, convective or advective-convective rainfall events over the Mediterranean basin. The number of monthly floods (blue bars) and flash-floods (black bars) is from Guzzetti et al.^101^, Gaume et al.^102^ and Diodato et al.^103^ for the period 1950–2006. For the same period, monthly percentiles (prc) were extracted from the CRU Global Climate Dataset^104^. The terms of Eq. (1) are reported.

The concept of the model is summarised in Fig. 7 following Waldam^95^ and Diodato and Bellocchi^96^. A separation of advective and convective events is considered important, assuming that convective precipitation (brief and intense) has higher dynamics and variability than the usually more static advective events. Seasonal differences in precipitation patterns are governed either by convective processes, i.e. higher dynamics more common in summer (when rain-splash dominate), or a variable mix of convective and advective rains more frequent in autumn (when diffuse overland flow dominates). Then, winter and spring rains (of long duration and low intensity), usually originating from broad mid-latitude frontal activity, carry high volumes of rainwater thanks to orographically enhanced stratiform precipitation causing large-scale hydrological processes like flooding^97^. High percentiles are used to differentiate more clearly such rainfall characteristics These processes are captured in the Rainfall Erosivity Mediterranean Model—Eq. (1)—by the storm-severity index (*ASSIS(GF)*). Highly intensive convective (or of a mixed advective-convective nature) rain events result instead in the splash-erosivity process, as interpreted by the variation coefficient $\frac{SD}{Max(Ps)}$. In this case, raindrops with high kinetic energy may cause local downpours.

### Trend assessment of extreme values

The quantile approach was applied to identify step trends at any *ED*_*X*_ scheme with assigned return periods. The return period (*T*) for the seasonal rainfall erosivity density falling above the jth quantile was ranked using the lognormal distribution:

5 $${Q\left({ED}_{Xt-w}\right)}_{T}=\mathrm{exp}\left[\upmu \left({ED}_{Xt-w}^{*}\right)+{u}_{T}\cdot \sigma \left({ED}_{Xt-w}^{*}\right)\right]$$

where ${Q\left({ED}_{Xt-w}\right)}_{T}$ is the *j*^th^ rainfall erosivity density (*ED*_*X*_) quantile of the log-normal distribution with assigned return period; $\upmu \left({ED}_{Xt-w}^{*}\right)$ and $\sigma \left({ED}_{Xt-w}^{*}\right)$ are the mean and the standard deviation of the variable *ED*_X t_* = ln(*ED*_*X t*_). The subscript *t*-*w* indicates the computation of the generic variable at the *t*-time over a 22-year moving window (*w*); *u*_*T*_ is the log-normal dimensionless coefficient equal to 1.27 for *T* = 30 years. The moving window of ~ 22-years reflects the periodicity of climate phenomena, which proved effective to show long-term trends by smoothing out the secular variation and the more volatile year-to-year changes^70^.

### Model calibration and assessment

To assess the model, statistical analyses were performed with STATGRAPHICS (http://www.duke.edu/~rnau/sgwin5.pdf), with the graphical support of WESSA (https://www.wessa.net) and CurveExpert routines (https://www.curveexpert.net). The parameters of Eq. 1 were calibrated against actual rainfall erosivity data according to statistical criteria. The first condition was to minimize the distance between modelled and actual erosivity data, by minimizing the Mean Absolute Error (optimum, 0 ≤ MAE < ∞, MJ mm hm^-2^ h^-1^ yr^-1^). Complementary to the MAE, the MAPE (mean absolute percent error) offers the advantage of being scale-independent and intuitive (e.g. the prediction model is considered reasonable with a MAPE below 30% and very accurate with a MAPE less than 10%). The second condition is to maximise the determination coefficient (0 ≤ R^2^ ≤ 1, optimum) that is the variance explained by the model. The third conditions approximates the unit slope of the straight line that would minimise the bias of the linear regression actual versus modelled data (b = 1, optimum). In addition, the Kling-Gupta index (–∞ < KGE ≤ 1) was used as efficiency measure, with KGE > –0.41 indicating that a model improves upon the means of observations as a benchmark predictor. The Nash–Sutcliffe efficiency (-∞ < EF ≤ 1, optimum) was also calculated as an uncertainty indicator of the model performance because greater values than 0.6 indicate limited model uncertainty, likely associated with narrow parameter uncertainty^98^. To select the set of important covariates for the parsimonious model for estimating actual erosivity data, we iteratively added in predictors, one-at-a-time until modelling solutions with small MAE and large R^2^ values were obtained. Then, for the final selection, the third criterion—|b-1|= min—was additionally involved. Each predictor was repositioned over > 50 iterations until convergence was achieved^99^. The Durbin-Watson statistic was performed to test for auto-correlated residuals because large temporal dependence may induce spurious correlations. ANOVA *p*-values were used to present the statistical significance of the regression between estimates and the actual data.

## Supplementary Information

Supplementary Information

## Data Availability

All data used in this study are freely available. Spatial patterns of mean annual rainfall erosivity over the European region (Fig. 1c) are freely available from the ESDAC (European Soil Data Centre) dataset at https://esdac.jrc.ec.europa.eu/content/global-rainfall-erosivity. Anomalies in the spatial pattern of annual mean rain rates over the Mediterranean region (Fig. 4d) are obtained from NCEP (US National Center for Environmental Prediction)/NCAR (US National Center for Atmospheric Research) reanalysis data at https://psl.noaa.gov/cgi-bin/data/getpage.pl. The proxy-based multi-decadal winter NAO reconstruction (Fig. 4a) is by Climate Explorer Climate Change Atlas of the Dutch Royal Netherlands Meteorological Institute (KNMI) at http://climexp.knmi.nl. Updated (R)USLE-based erosivity data (Fig. 5) were derived from the system for the collection and elaboration of climatological data (SCIA) of the Italian National Institute for Environmental Protection and Research (ISPRA) at http://www.scia.isprambiente.it/wwwrootscia/scia.html, completed with the data provided from the digital National Agrometeorological Network accessible through https://tinyurl.com/h4juzuv. Updated seasonal precipitation datasets were retrieved from the Global Precipitation Climatology Centre (GPCC) through https://tinyurl.com/wlnvznq. Also, the full set of raw data and the equations that support the findings of this study (erosivity model, time-series reconstruction and precipitation fraction data, seasonal precipitation and storm-severity index inputs), are available in the supplementary Table S1.
